# Prediction for Intravenous Immunoglobulin Resistance Combining Genetic Risk Loci Identified From Next Generation Sequencing and Laboratory Data in Kawasaki Disease

**DOI:** 10.3389/fped.2020.462367

**Published:** 2020-12-04

**Authors:** Liqin Chen, Sirui Song, Qianqian Ning, Danying Zhu, Jia Jia, Han Zhang, Jian Zhao, Shiying Hao, Fang Liu, Chen Chu, Meirong Huang, Sun Chen, Lijian Xie, Tingting Xiao, Min Huang

**Affiliations:** ^1^Department of Cardiology, Shanghai Children's Hospital, Shanghai Jiao Tong University, Shanghai, China; ^2^Shanghai Center for Bioinformation Technology, Shanghai, China; ^3^Department of Cardiothoracic Surgery, Stanford University School of Medicine, Stanford, CA, United States; ^4^Clinical and Translational Research Program, Betty Irene Moore Children's Heart Center, Lucile Packard Children's Hospital, Palo Alto, CA, United States; ^5^Heart Center, Children's Hospital of Fudan University, Shanghai, China; ^6^Department of Cardiology, Shanghai Children's Medical Center Affiliated to Shanghai Jiao Tong University, Shanghai, China; ^7^Department of Pediatric Cardiology, Xinhua Hospital, Affiliated to Shanghai Jiao Tong University School of Medicine, Shanghai, China

**Keywords:** Kawasaki disease, intravenous immunoglobulin resistance, gene, laboratory data, predictive model

## Abstract

**Background:** Kawasaki disease (KD) is the most common cause of acquired heart disease. A proportion of patients were resistant to intravenous immunoglobulin (IVIG), the primary treatment of KD, and the mechanism of IVIG resistance remains unclear. The accuracy of current models predictive of IVIG resistance is insufficient and doesn't meet the clinical expectations.

**Objectives:** To develop a scoring model predicting IVIG resistance of patients with KD.

**Methods:** We recruited 330 KD patients (50 IVIG non-responders, 280 IVIG responders) and 105 healthy children to explore the susceptibility loci of IVIG resistance in Kawasaki disease. A next generation sequencing technology that focused on 4 immune-related pathways and 472 single nucleotide polymorphisms (SNPs) was performed. An R package SNPassoc was used to identify the risk loci, and student's *t*-test was used to identify risk factors associated with IVIG resistance. A random forest-based scoring model of IVIG resistance was built based on the identified specific SNP loci with the laboratory data.

**Results:** A total of 544 significant risk loci were found associated with IVIG resistance, including 27 previous published SNPs. Laboratory test variables, including erythrocyte sedimentation rate (ESR), platelet (PLT), and C reactive protein, were found significantly different between IVIG responders and non-responders. A scoring model was built using the top 9 SNPs and clinical features achieving an area under the ROC curve of 0.974.

**Conclusions:** It is the first study that focused on immune system in KD using high-throughput sequencing technology. Our findings provided a prediction of the IVIG resistance by integrating the genotype and clinical variables. It also suggested a new perspective on the pathogenesis of IVIG resistance.

## Introduction

Kawasaki disease (KD) is an acute systemic vasculitis of unknown origin, and has been the leading cause of pediatric acquired heart disease. The diagnosis of KD is based on the presence of fever for at least 5 days and 5 clinical criteria including rash, bilateral bulbar conjunctival injection, erythema of oral and pharyngeal mucosa, erythema and edema of the hands and feet, and cervical lymphadenopathy (1). The primary treatment for Kawasaki disease is intravenous immunoglobulin (IVIG). However, there were 10–20% of patients who were resistant to IVIG, causing an increased risk of coronary artery lesions (CALs) (2).

A few scoring systems were developed to predict IVIG resistance in patients with KD. Kobayashi scoring model was constructed, including day of illness at initial treatment, age in months, percentage of white blood cells representing neutrophils, platelet count, and serum aspartate aminotransferase, sodium, and C-reactive protein, which yielded a sensitivity of 86% and specificity of 68% for a cut-off point four or more (3). The sensitivity of the Kobayashi scoring model, however, was low (31.4–57.1%) when validated on patients in Korea, North America, and China (3–5). One potential reason is the variations of genes and environments in different population, indicating the necessity of including the genetic factors into the IVIG resistance prediction. A variety of genetic markers associated with IVIG unresponsiveness and CAL formation in KD patients were found by Genome-wide association studies (GWAS), including *inositol 1,4,5-trisphosphate 3-kinase C* (*ITPKC*), *caspase-3* (*CASP3*), *FCGR2A, CD40*, and *interleukin1 beta* (*IL-1B*) (6–9).

Our study aims to develop a model to provide a prediction of the IVIG resistance for patients with KD in China. A low-cost, high-accuracy targeted capture sequencing technology was used to identify genes and SNP loci associated with immune system in KD. The initial targeted capture goal area included 560 genes and 472 SNPs susceptibility loci reported in previous KD studies. The identified genes were involved in T cell receptors signaling pathways, transforming growth factor-beta (TGF-β) signal pathway, toll-like receptors signaling pathways and cytokine receptors. Using the identified genes, SNPs, as well as clinical variables, a scoring model was built to evaluate the risk of IVIG resistance of KD patients.

## Methods

### Study Population

Patients who were diagnosed as KD and received IVIG treatment at Shanghai Children's Hospital from 2015 to 2018 were recruited. The diagnosis criteria of KD were based on the guidelines proposed by the American Heart Association in 2017 (1). Children were diagnosed as KD who had the presence of fever for at least 5 days and fulfilled four of following clinical features:erythema of mucosa with strawberry tongue and cracking lips, bilateral non-purulent conjunctivitis, dysmorphic skin rashes, erythema, and edema of the hands and feet in acute phase or periungual desquamation in subacute phase and unilateral cervical lymphadenopathy. All the patients were treated with high dose IVIG (2 g/kg) within 10 days of the onset of the disease. Aspirin (3–5 mg/kg/day) was given until inflammation was resolved or no evidence of coronary changes existed under 2-dimensional echocardiography test. CAL was defined by the increment of internal diameter of 3 mm (≤5 years old) or 4 mm (>5 years old), or 1.5 times larger of internal diameter than the adjacent segment, according to the guidelines of Japanese Ministry of Health (10). KD patients who had recurrent or persistent fever within 36 h after the end of IVIG treatment were defined as IVIG non-responders. This study has been reviewed and approved by the ethics committee of children's hospital affiliated to Shanghai Jiao Tong University (2017R034-E02). The informed consent was obtained by the parents or their guardians.

### Clinical Data Capturing

Clinical data of patients who visited the Shanghai Children's Hospital were entered into a database called Clinical Information System (CIS) on a daily basis. In this study, electronic medical records of KD patients were identified and extracted from the CIS using Doctor Research Information Management (DRIM) system, and a structured database was built to support the following analyses (11).

### DNA Extraction and Targeted Enrichment of Genomic Region Technology

Blood samples of each KD patient were collected by the EDTA anticoagulation tube. DNA was extracted using Genomic DNA Extraction Kit (Cat. No.DP329, TIANGEN Bioscience Beijing), and the library was constructed using KAPA HTP Library preparation kit (Cat. No. KK8234, Roche, USA). Illumina HiSeq X10 (Illumina USA) was used as the sequencing platform for libraries target enriched by custom capture array. The hybridization probes of the custom capture array consisted of previous published 472 GWAS hotspots related to KD and 560 candidate genes among 4 KEGG pathways including Toll-like receptor signaling pathway, Cytokine receptor interaction, TGF-β signaling pathway and T cell receptor signaling pathway. The targeted capture chips were designed by SeqCap EZ Choice (Roche, Switzerland).

### Statistical Analysis

The raw data were mapped to the human reference genome (hg19) using the Burrows-Wheeler alignment (BWA v0.7.15) tool (12) and Picard tool (v1.135) was used to process for PCR duplicates (http://broadinstitute.github.io/picard/). Candidate SNPs were detected using GATK (v3.7) HaplotypeCaller algorithm (13), and filtered with following parameters “QD <2.0 || FS >60.0 || MQ <40.0 || MQRankSum < -12.5 || ReadPosRankSum < -8.0.” Variants annotation was performed using ANNOVAR (14), and genetic risk-alleles associated IVIG resistance were analyzed using R package SNPassoc (15). All statistical analyses were performed in R. Student's *t*-test was used to compare the differences of clinical features between the IVIG non-responders and IVIG responders. *P* < 0.05 was considered statistically significant. The scoring model of IVIG resistance was developed based on the identified specific SNP loci as well as the laboratory data. R package ipred was used to train the model (16).

### Weighted Genetic Risk Scoring System

The cumulative effects of candidate SNPs were calculated though wGRS system designed by De Jager et al. (17). The wGRS was calculated by multiplying the weight by the risk allele number (0, 1, or 2), and taking the sum across SNPs:

$$\begin{array}{l} wGRS=\overset{n}{\underset{k=1}{\sum }}w^{k}G^{k} \end{array}$$

where n is the number of SNP, k is SNP, w^k^ is the corresponding weight of SNP [ln(OR)], and G^k^ is the number of the risk allele (0, 1, or 2). wGRS was compared between the IVIG responders and non-responders using the Wilcoxon rank-sum test with continuity correction. The performance of the model was evaluated by the area under the receiver operating characteristic (ROC) curve.

## Results

### Clinical Characteristics of IVIG Non-responders

A total of 330 KD patients were enrolled in this study, with 50 (15.2%) IVIG non-responders and 280 (84.8%) IVIG responders. The male to female ratio was 1.77:1.0 (211:119). All the patients received IVIG treatment (2 g/kg) and aspirin with 3–5 mg/kg/day. Treatment was repeated on 50 patients due to the IVIG resistance. Forty-eight patients were ultimately found with CAL, and 15 of the 48 patients showed IVIG resistance.

There was no significant difference in gender and age distribution between IVIG responders and non-responders (Table 1). Consistent with previous studies of the epidemiology and presentation of KD, the majority of patients were male [76% (38 of 50) in IVIG non-responders and 62% (173 of 280) in IVIG responders]. Though IVIG non-responders were younger (30.5 ± 24.5 months) than IVIG responders (34.3 ± 27.3 months), the difference was not significant (*P* > 0.05).

**Table 1:** Patients diagnose and characteristics.

	**No. of patients**	**No. of male**	**No. of female**	**Age, month, mean ± SD**
IVIG-non-response	50	38	12	30.5 ± 24.5
IVIG-response	280	173	107	34.3 ± 27.3

**Figure 1:**
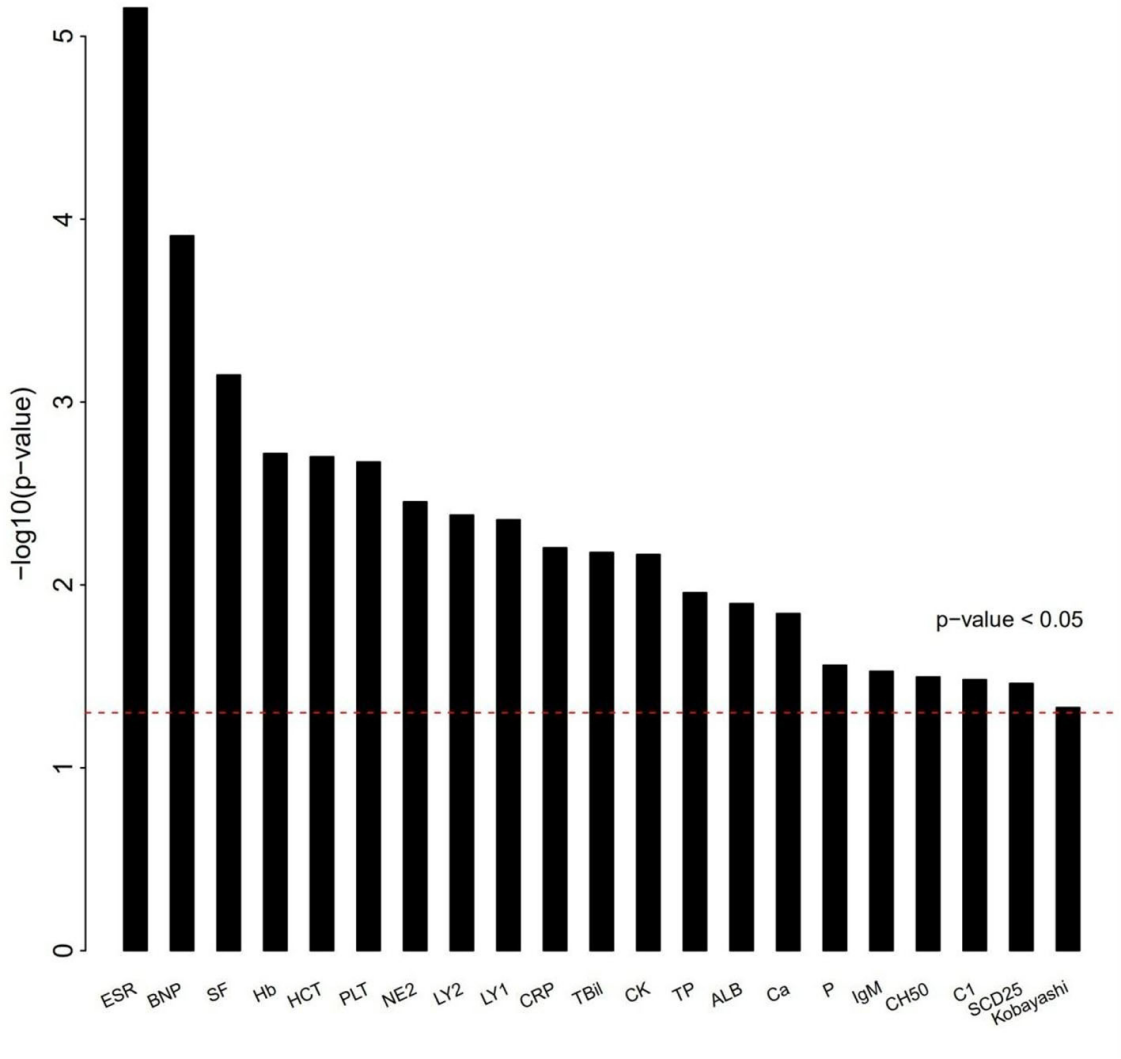
Differences of clinical characteristics between IVIG unresponders and responders. Clinical variables as *x* axis and *P*-value of clinical variables with *y* axis. The clinical variables were different significantly between IVIG unresponders and responders (*P* < 0.05).

The differences of clinical features that may be related to IVIG resistance between two groups were calculated (Figure 1). As shown in Table 2, 6 clinical variables met the condition of *P* < 0.05 and percentage of patients with adequate data <30% were found. Compared with the subjects who were responsive to the IVIG therapy, IVIG non-responders were more likely to have higher C-reactive protein level, higher percentage of neutrophils, lower platelet count, lower serum albumin concentration, lower erythrocyte sedimentation rate, and lower hemoglobin level. Patients with IVIG resistance had longer fever duration (Table 2).

**Table 2:** Clinical indicators of IVIG non-responders and IVIG responders.

**Clinical indicators**	**IVIG non-responders**	**Patients with adequate data, %**	**IVIG responders**	**Patient with inadequate data, %**	***P*-value**
Fever days	11.91 ± 2.96	10	7.79 ± 2.96	2.86	2.0 × 10^−5^
PLT (× 10^9^/L)	315.46 ± 121.27	12	361.38 ± 121.27	3.93	3.19 × 10^−2^
CRP (mg/L)	103.78 ± 46.04	14	65.59 ± 46.04	3.57	2.0 × 10^−5^
ALB (g/L)	33.62 ± 3.86	16	36.22 ± 3.86	6.07	2.1 × 10^−3^
ESR (mm/h)	71.15 ± 29.13	18	88.24 ± 29.13	7.86	6.6 × 10^−3^
Hb (g/L)	105.81 ± 10.59	20	109.55 ± 10.59	13.21	2.75 × 10^−2^
NE (%)	72.96 ± 15.69	28	64.82 ± 15.69	3.93	4.1 × 10^−3^
HCT (%)	31.36 ± 2.86	30	32.51 ± 2.86	4.29	1.43 × 10^−2^
LY (%)	19.85 ± 13.06	30	26.17 ± 13.06	3.93	1.27 × 10^−2^
LYM (×10^9^/L)	2.62 ± 2.05	30	3.52 ± 2.05	4.64	3.5 × 10^−3^
TP (g/L)	59.00 ± 6.29	32	63.37 ± 6.29	6.43	1.9 × 10^−3^
TBil (μmol/L)	18.56 ± 12.61	40	9.80 ± 12.61	16.07	3.30 × 10^−2^
CK (U/L)	34.59 ± 66.16	42	56.73 ± 66.16	17.86	1.0 × 10^−4^
Kobayashi	2.59 ± 1.58	42	1.54 ± 1.58	17.14	6.3 × 10^−3^
Ca (mmol/L)	2.22 ± 0.12	50	2.29 ± 0.12	25.00	2.97 × 10^−2^
P (mmol/L)	1.07 ± 0.28	50	1.30 ± 0.28	25.36	2.0 × 10^−3^
SF (ng/ml)	218.00 ± 90.26	56	149.99 ± 90.26	34.29	3.46 × 10^−2^
BNP (pg/ml)	2975.17 ± 1210.82	60	782.36 ± 1210.82	36.79	1.1 × 10^−2^
IL-2R (pg/ml)	84383.11 ± 48547.54	72	43036.82 ± 48547.54	48.93	4.69 × 10^−2^
IgM (g/L)	0.84 ± 0.49	74	1.12 ± 0.49	63.57	4.4 × 10^−3^
CH50 (μ/ml)	50.19 ± 9.37	78	58.98 ± 9.37	63.93	7.0 × 10^−4^
C1 (mg/L)	173.67 ± 53.43	80	207.37 ± 53.43	2.86	6.8 × 10^−3^

*PLT, platelet count; CRP, C-reactive protein; ALB, albumin; ESR, erythrocyte sedimentation rate; Hb, Hemoglobin; NE%, percentage of neutrophils; HCT, Hematocrit; LY%, percentage of lymphocyte; LYM, lymphocyte absolute value; TP, total protein; TBIL, total bilirubin; CK, creatine kinase; Ca, serum calcium; P, serum phosphorus; SF, serum ferritin; BNP, brain natriuretic peptide; IL-2R, interleukin-2 receptor; IgM, Immunoglobulin M; CH50, 50% of complement hemolytic activity; C1, complement 1*.

*P-value for continuous variables from t-test*.

### Risk Loci Affecting IVIG Unresponsiveness

To identify the gene variants associated with IVIG resistance, 330 patients diagnosed as KD and 105 healthy children were recruited in this phase. A second-generation sequencing focusing on the exon regions of 560 genes in four immune related pathways and 472 KD-associated SNPs reported in previous GWAS studies were conducted. The average sequencing depth of KD samples and normal samples were 477X and 445X, respectively. A total of 15,677 SNP sites were found in all samples. Genotypes of 544 SNPs in targeted regions were significantly different (*P* < 0.05) between IVIG responders and non-responders. The common variants were identified that the minor allele frequency (MAF) of the gene or SNP was more than 1%.

There were 14 SNPs (MAF >1%) that showed significant difference between IVIG non-responders and IVIG responders (*P* < 0.001; Table 3). Among all identified risk loci, SNP rs840016 that located at the region of *CD247* showed the largest correlation (P = 5.4 × 10^−4^). An increasing risk of IVIG resistance was observed (odd ratio = 3.77) by comparing the risk allele (T allele) to the non-risk allele (C allele). Another SNP rs2463260 A/G (*P* = 4.2 × 10^−4^) located in chromosome 1 was filtered out with an odd ratio 3.35, which risk allele was G. rs56401579G/A and rs56193546C/T located at *FLT4* gene region were also related with IVIG unresponsiveness, which odd ratios were 3.33 when risk alleles compared with non-risk alleles. The minor alleles of rs10271133C/T and rs2007404T/C that located in *CUL1* gene region showed high incidence of IVIG resistance. Moreover, transformation of rs77317995T/A located in *ACVR2B* gene region from major allele to minor allele was associated with protection from IVIG resistance. rs6530599A/G, rs6530600A/G, and rs1136210A/G located on chromosome Y (*CD24*) were found to be protective gene associated with IVIG unresponsiveness (*P* = 7.0 × 10^−4^, odds ratio, 0.55). The frequencies of AA, AG, and GG genotypes were 17.1, 82.5, and 0.5% in males, while that were 16.0%, 0.8%, and 83.2% in females. Besides, rs2232595C/T, rs16949924G/C, rs12358961T/A, and rs742185A/G were found association with IVIG unresponsiveness.

**Table 3:** 14 SNP associated with IVIG unresponsive in KD.

**CHR**	**Position**	**SNP**	**Nearby Gene(s)**	**Region**	**Ref/Alt**	**Freq**	**OR**	***P*-value**	**Adjusted by gender**	**Adjusted by age**	***P*-value adjusted by age and gender**
1	40,229,368	rs2463260	*PPIE*	Exonic	A/G	0.06	3.35	4.2 × 10^−4^	4.6 × 10–^4^	1.6 × 10^−2^	1.5 × 10^−2^
1	167,408,670	rs840016	*CD247*	Intronic	C/T	0.02	3.77	5.4 × 10^−4^	5.4 × 10^−3^	1.0 × 10^−1^	1.33 × 10^−1^
3	38,534,142	rs77317995	*ACVR2B*	UTR3	T/A	0.36	0.46	4.4 × 10^−4^	4.2 × 10^−4^	1.3 × 10^−2^	1.2 × 10^−2^
5	180,053,090	rs56401579	*FLT4*	Intronic	G/A	0.04	3.33	3.6 × 10^−4^	5.4 × 10^−4^	3.2 × 10^−3^	4 × 10^−3^
5	180,053,097	rs56193546	*FLT4*	Intronic	C/T	0.04	3.33	3.6 × 10^−4^	5.4 × 10^−4^	3.2 × 10^−3^	4 × 10^−3^
7	148,480,990	rs10271133	*CUL1*	Intronic	C/T	0.05	3.58	8 × 10^−5^	8.2 × 10^−5^	6.5 × 10^−3^	5 × 10^−3^
7	148,487,395	rs2007404	*CUL1*	Intronic	T/C	0.11	2.62	8.8 × 10^−4^	9.2 × 10^−4^	1.4 × 10^−1^	1.4 × 10^−1^
10	6,066,195	rs12358961	*IL2RA*	Intronic	T/A	0.07	2.94	4.7 × 10^−4^	5.5 × 10^−4^	1.2 × 10^−1^	1.4 × 10^−1^
15	66,727,597	rs16949924	*MAP2K1*	Intronic	G/C	0.05	2.97	8.8 × 10^−4^	9.7 × 10^−4^	2.2 × 10^−1^	2.21 × 10^−1^
20	36,989,335	rs2232595	*LBP*	Intronic	C/T	0.02	3.74	9.6 × 10^−4^	6.6 × 10^−4^	2.1 × 10^−3^	2 × 10^−3^
22	50,705,059	rs742185	*MAPK11*	Intronic	A/G	0.09	2.87	5.1 × 10^−4^	5.1 × 10^−4^	3.0 × 10^−1^	3.02 × 10^−1^
Y	21,153,275	rs6530599	*CD24*	ncRNA_intronic	A/G	0.57	0.55	7 × 10^−4^	8.4 × 10^−4^	3.6 × 10^−1^	3.66 × 10^−1^
Y	21,153,459	rs6530600	*CD24*	ncRNA_intronic	A/G	0.57	0.55	7 × 10^−4^	8.4 × 10^−4^	3.6 × 10^−1^	3.66 × 10^−1^
Y	21,153,474	rs1136210	*CD24*	ncRNA_intronic	A/G	0.57	0.55	7 × 10^−4^	8.4 × 10^−4^	3.6 × 10^−1^	3.66 × 10^−1^

*Ref/Alt, reference allele and alternative allele; OR, indicates odds ratio; and UTR, untranslated region*.

### wGRS Analysis and ROC Curve Analysis

To detect the diagnostic accuracy of clinical evaluations of IVIG treatment for KD patients using candidate SNPs, a wGRS system was applied (17). Four hundred and forty eight SNPs (MAF >1%) that reached suggestive significance were identified between IVIG non-responders and IVIG responders (*P* < 0.05). To identify more effective predictors, the risk loci between IVIG resistant group and healthy control group were evaluated, where 198 SNPs (MAF >1%) were found significantly different. Nine SNPs were identified to be rick loci associated with IVIG resistance by comparing 488 SNPs and 198 SNPs, and were used to build a scoring model (Supplementary Table 1).

**Figure 2:**
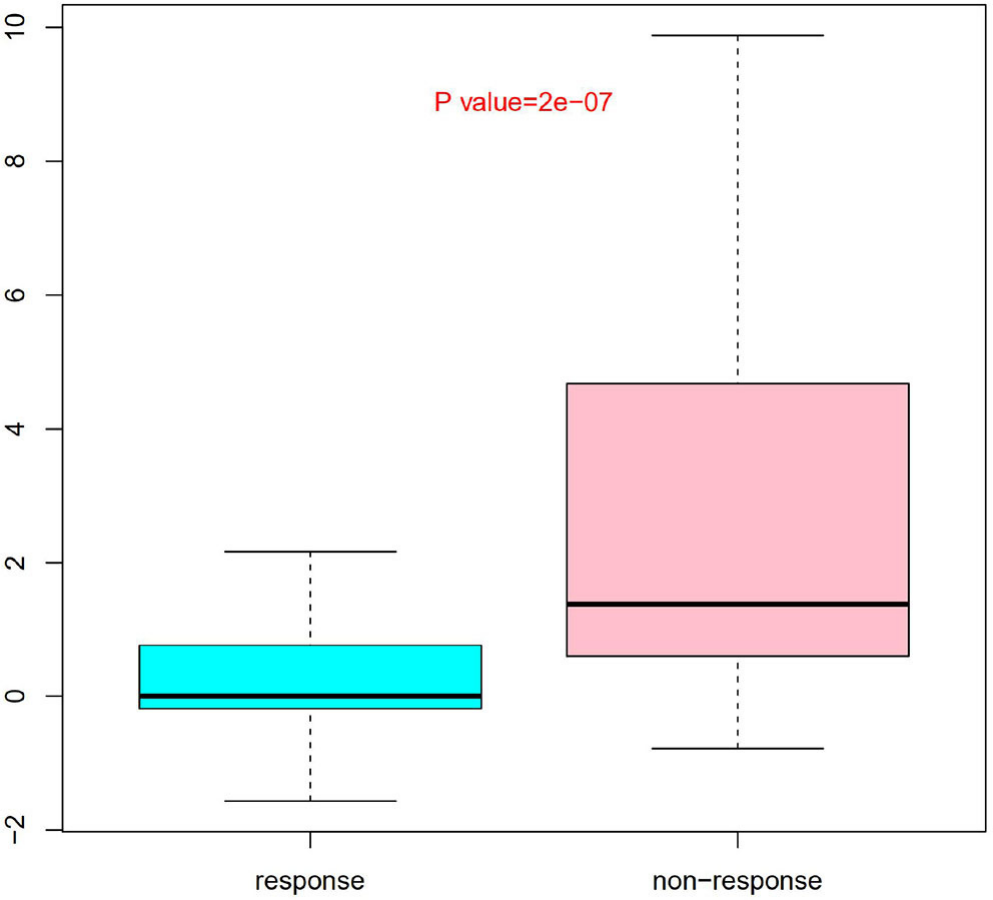
Evaluation difference of weighted genetic risk score (wGRS) of 9 SNPs between IVIG non-responders and responders. Group as *x* axis and wGRS scores of 9 SNPs with *y* axis. The difference of wGRS scores between the two groups was significant (Wilcoxon rank-sum test, *P* = 2.0 × 10^−7^).

**Figure 3:**
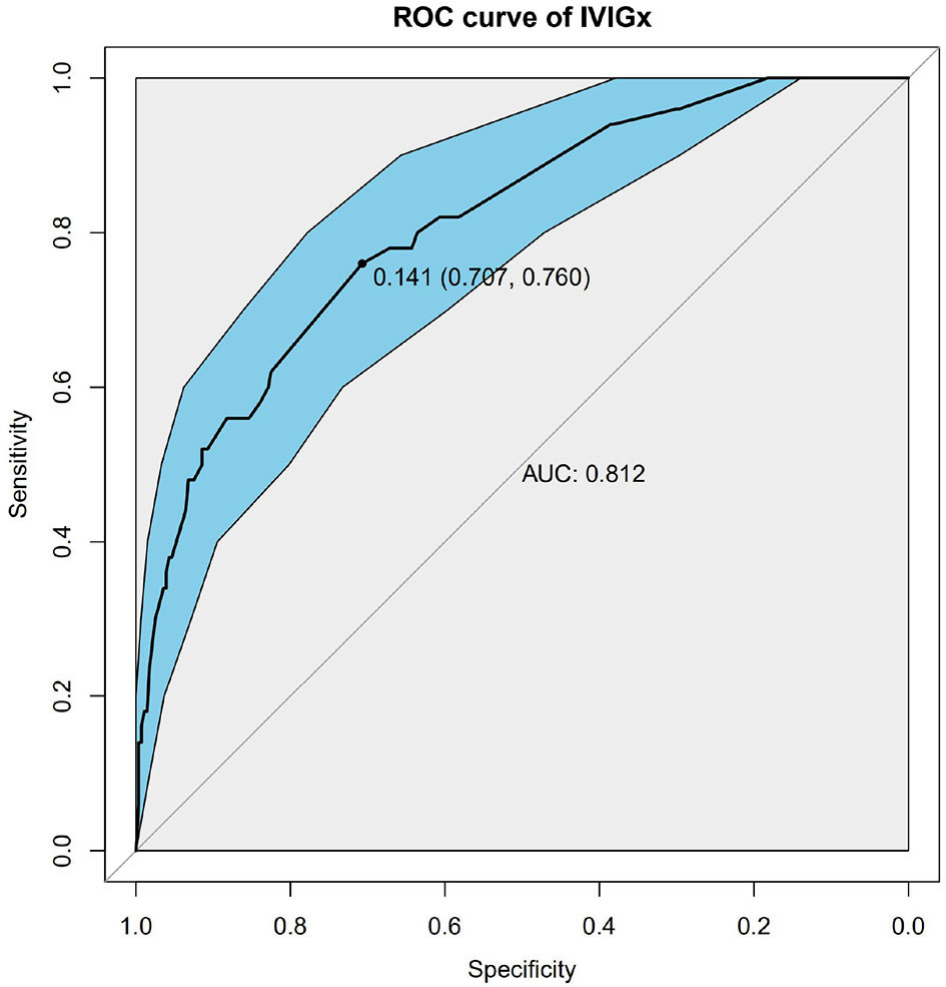
Receiver operating characteristic (ROC) curve analysis of the model using weighted genetic risk score (wGRS) of 9 SNPs to predict IVIG resistance in KD. ROC of wGRS with Specificity as *x* axis and sensitivity with *y* axis. The most predictive wGRS value (0.141) and the corresponding specificity and sensitivity (76 and 70.7%) were shown.

**Figure 4:**
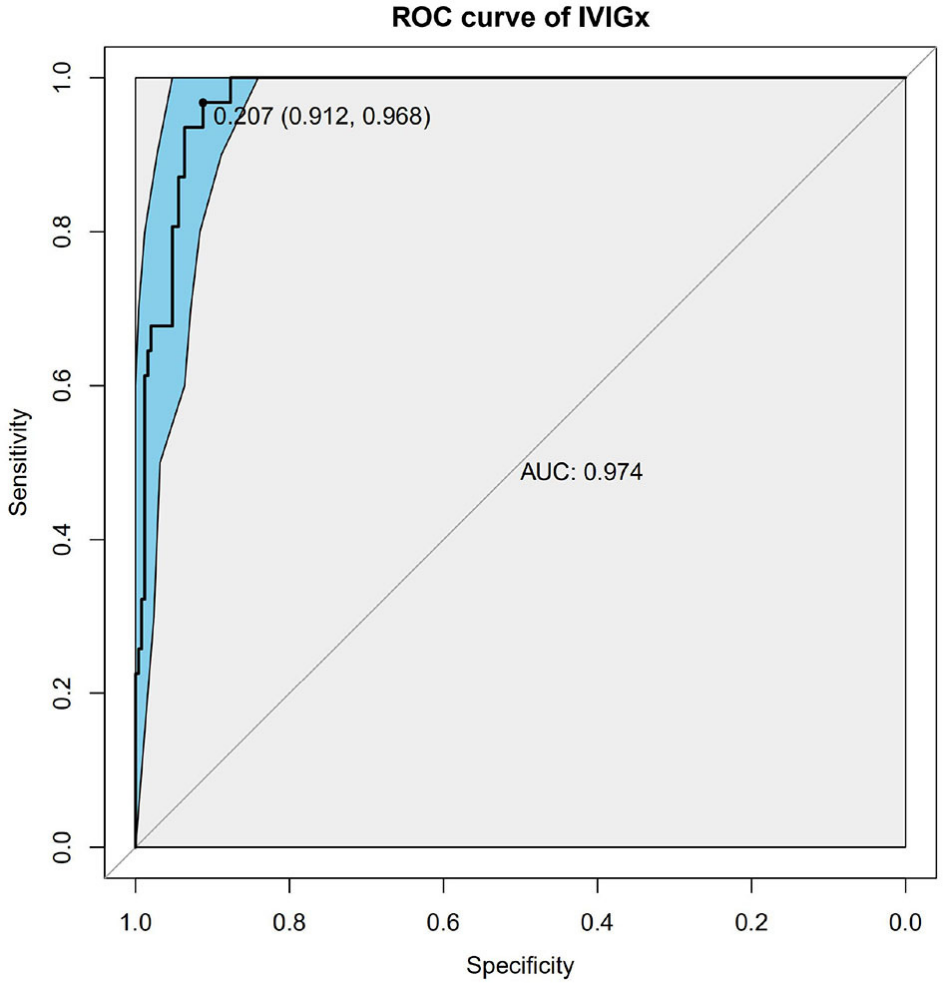
Receiver operating characteristic (ROC) curve analysis of the combinatory model based on weighted genetic risk score (wGRS) of 9 SNPs and clinical variables. ROC of wGRS with Specificity as *x* axis and sensitivity with *y* axis. The most predictive wGRS value (0.207) and the corresponding specificity and sensitivity (91.2 and 96.8%) were shown.

Nine SNPs (Supplementary Table 1) with *P* < 3 × 10^−3^ were included in the wGRS algorithm. None of two SNPs out of 9 showed strong linkage disequilibrium. The difference of wGRS scores between the two groups was significant (Wilcoxon rank-sum test, *P* = 2.0 × 10^−7^) (Figure 2). The scoring model which based on wGRS score of 9 SNPs yielded a sensitivity of 76% and specificity of 70.7% for a cutoff point of 0.141 (Figure 3). C-reactive protein level, percentage of neutrophils, platelet count, serum albumin concentration, erythrocyte sedimentation rate, and hemoglobin level were identified with highest risk for IVIG unresponsiveness. The six clinical variables and wGRS score of 9 SNPs were used to generate a composite scoring model which revealed better sensitivity (96.8%) and specificity (91.2%) for a cutoff point of 0.207 (Figure 4).

We also tested Kobayashi scoring system to predict KD patients who resistant to IVIG. The sensitivity and specificity were 34.5 and 88% in the Kobayashi scoring system.

## Discussion

In this study, we established a novel scoring model to predict IVIG resistance in KD. Patients with 6 abnormal clinical variables (C-reactive protein level, percentage of neutrophil, platelet count, serum albumin concentration, erythrocyte sedimentation rate, and hemoglobin level) were more likely to be resistant to IVIG therapy. The wGRS score of SNPs were effective in identifying IVIG resistance.

T-cell receptor zeta chain (CD3ζ) encoded by *CD247* is a component of the T-cell receptor (TCR) complex. CD3ζ chain can alter TCR signaling and participate in T cell activation (18). Previous studies found that CD247 were associated with Systemic lupus erythematosus (SLE) and other autoimmune disorders by T cell-mediated mechanism (19, 20). Interleukin-2 receptor subunit alpha encoded by *IL2RA* gene is a hallmark antigen of regulatory T cells, and functions in the suppression of immune responses and maintenance of immune homeostasis (21, 22). Kuo et al. (23) reported that *IL2RA* (rs3118470) was significantly associated with CAL formation in KD patients. We found that SNP rs840016 C/T located at *CD247* and rs12358961 T/A located at *ILR2A* have association with the increasing risk of IVIG resistance.

Lipopolysaccharide binding protein (LBP) is a phase protein that is synthesized mainly in the liver and binds lipopolysaccharide (LPS) to initiate the immune response. LPS is presented to cluster of differentiation (CD) 14, which interacts with toll-like receptor to activate the immune (24). A study about postoperative patients with sepsis, LBP plasma concentration showed similar course with CRP in predicting outcome (25). The fms related tyrosine kinase (FLT4) is known as the vascular endothelial growth factor receptor (VEGFR) 3, which is a protein kinase and regulates endothelial cell growth and angiogenesis (26). Some study reported that the increased serum level of VEGF had associated with serum levels of CRP in acute phase of KD (27). The level of CRP was higher in IVIG un-responders than IVIG responders in our study. We also found *LBP* and *FLT4* gene variants were positively correlated with IVIG treatments.

The MAPK pathway is a signal transduction pathway and regulates fundamental cell activities including proliferation, transcriptional regulation, differentiation and survival. Mitogen-activated protein kinases (MAPK) is activated by TGF-β1 that induces endothelial cell apoptosis. MAPK 11 (p38β) is the predominant form of MAPK (28). He et al. (29) reported that MAPK11 mediated p38 activity was associated with osteolytic bone destruction in breast cancer cells. Zhang et al. (30) reported the oncogenic mutations within the β3-αC loop of *MAP2K1* have profound effect on drug response and can be critical for targeted therapies. Activin A receptor type 2B (ACVR2B) is activated by the activin family of ligands of TGF-β superfamily and activate SMAD2/3 signaling pathway (31). Genetic variants in TGF-β/SMAD3 signaling pathway were consistently associated with KD susceptibility, CAL and IVIG therapy response (32). We found that patients with genetic variants of *MAPK11* and *MAP2K1* had higher risk of IVIG unresponsiveness. The mechanism of *MAPK11 and MAP2K1* associated with IVIG resistance may participate in the TGF-β/SMAD3 signaling pathway. It is interesting that rs77317995T/A located in gene *ACVR2B* may be the protective factor of IVIG treatment in our study.

CD24 is a phosphatidylinositol (GPI)-linked protein expressed on the cell surface that have been implicated in the stimulation of T cell and differentiation of B cells. *CD24* gene is a member of multigene family and have homologous sequences on chromosome 6 and chromosome. Lee et al. (33) reported that high expression of CD24 can reduce inflammatory response by regulating NFκB in juvenile human chondrocytes. KD was found affects males 1.5 times more than female (34). We reported that three SNPs located at *CD24* on chromosome Y were associated with IVIG resistance. The frequency of risk allele A may explain the predominance of males in IVIG resistant group.

Cullin1 (CUL1) is the first and most extensively characterized member of the cullin family and an essential component of SCF E3 ubiquitin ligase complex. A study has reported that the expression of CUL1 in colorectal cancer (CRC) can be valuable molecular markers to predict the prognosis of the CRC patients (35). Huang et al. (36) proposed that CUL1 is associated with the disease-specific survival of the breast cancer and may serve as a therapeutic target for breast cancer metastasis. In our study, there are two SNPs located at *CUL1* which associated with KD and can predict IVIG resistance.

Kuo et al. (37) first applied wGRS analysis to KD patients for IVIG responsiveness evaluation based on 11 single-nucleotide polymorphisms. The specificity and sensitivity of the scoring model were 81.7 and 79.2% after adjusted for sex effects. We then aggregated laboratory characteristics and genetic variations to build a scoring system for IVIG resistance prediction. We found that high C-reactive protein, high percentage of neutrophils, and low albumin were independent risk factors for IVIG resistance. Compared with the Kobayashi score, a predictor of IVIG resistance and CAL development based on Japanese patients, the sensitivity of our proposed model was higher (sensitivity 76–96.8%). Our findings were consistent with a study from UK that reported a failure of Kobayashi score in identifying IVIG resistance (38). Berdej-Szczot et al. (39) also found a reduced accuracy of Kobayashi score in predicting IVIG resistance in Poland. When Lin et al. (40) applied the Kobayashi score to predict the effect of IVIG therapy for KD patients in China Taiwan, they found that the sensitivity and specificity of the model were only 62 and 71%. Gene indicators can improve the accuracy of score system to predict IVIG unresponsive for KD patients, and these genetic variations together with the clinical indicators might suggest the development of disease and outcome of the therapy.

The study had several limitations. (1) We could not evaluate the new scoring model in other cohort of KD patients because the measurement of gene were not routine at other medical institution. (2) This was a single medical center, selection bias might occur such as the lack of adequate data of KD patients.

## Conclusions

We developed a predicting scoring system based on both clinical variables and gene variants. Using target enrichment of genomic region technology, several SNPs were found significantly different between IVIG responders and non-responders. The proposed model based on the wGRS score of SNPs and clinical features showed a high sensitivity, and can be implemented to predict IVIG resistance for KD patients.

## Data Availability Statement

This article contains previously unpublished data. The name of the repository and accession number are not available.

## Ethics Statement

This study was carried out in accordance with the ethics committee of children's hospital affiliated to Shanghai Jiao Tong University of guidelines, ethics committee of children's hospital affiliated to Shanghai Jiao Tong University with written informed consent from all subjects. All subjects gave written informed consent in accordance with the Declaration of Helsinki. The protocol was approved by the ethics committee of children's hospital affiliated to Shanghai Jiao Tong University.

## Author Contributions

MiH and TT contributed to conception and design of the study. LX, CC, FL, MeH, and SC contributed to the manuscript revision. SS, JJ, JZ, and SH performed data analysis. LC, QN, and HZ wrote the draft of manuscript. All authors contributed to the article and approved the submitted version.

## Conflict of Interest

The authors declare that the research was conducted in the absence of any commercial or financial relationships that could be construed as a potential conflict of interest.
