# Supplementary material for: Prediction for Intravenous Immunoglobulin Resistance Combining Genetic Risk Loci Identified From Next Generation Sequencing and Laboratory Data in Kawasaki Disease
Source: Front Pediatr. 2020 Dec 4;8:462367. doi: 10.3389/fped.2020.462367 (PMC7746618; doi:10.3389/fped.2020.462367)
Supplement: Supplementary file 1 [file Data_Sheet_1.PDF]

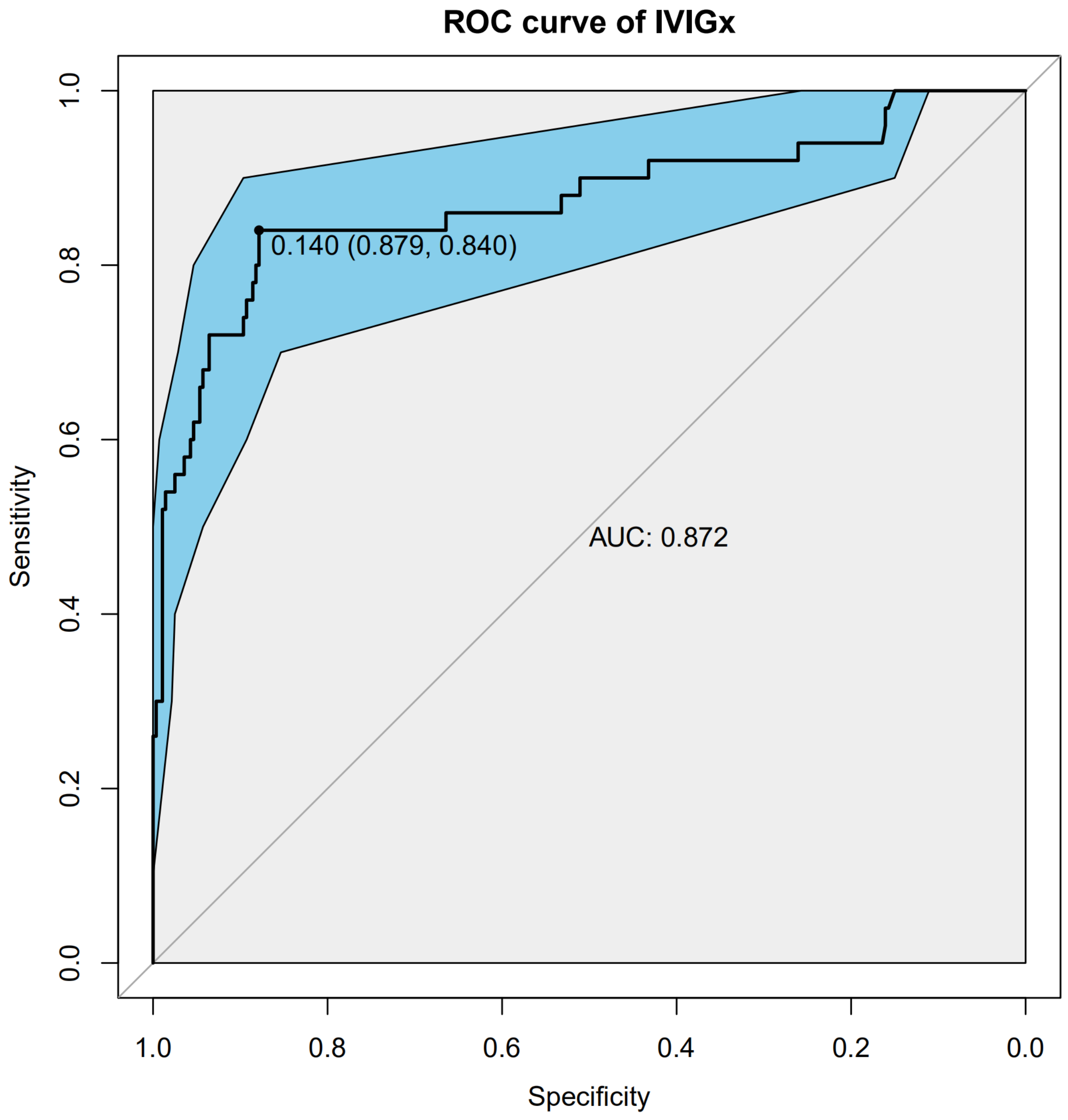

Supplementary Figure1 | Receiver operating characteristic (ROC) curve analysis of the model connected weighted genetic risk score (wGRS) of SNP with clinical characterics to predict IVIG resistance including incomplete data.
