# Supplementary material for: Prediction for Intravenous Immunoglobulin Resistance Combining Genetic Risk Loci Identified From Next Generation Sequencing and Laboratory Data in Kawasaki Disease
Source: Front Pediatr. 2020 Dec 4;8:462367. doi: 10.3389/fped.2020.462367 (PMC7746618; doi:10.3389/fped.2020.462367)
Supplement: Supplementary file 2 [file Table_1.DOCX]

**Supplementary Table1**｜9 SNPs used to build predicting IVIG unresponsive model

| CHR | Position | SNP | Nearby Gene(s) | Region | Ref/  Alt | Freq | OR | P-value |
| --- | --- | --- | --- | --- | --- | --- | --- | --- |
| chr1 | 52703169 | rs41309181 | ZFYVE9 | exonic | T/C | 0.05 | 2.59 | 1.6×10^-4^ |
| chr3 | 38534142 | rs77317995 | ACVR2B | UTR3 | T/A | 0.36 | 0.46 | 4.35×10^-4^ |
| chr7 | 148487395 | rs2007404 | CUL1 | intronic | T/C | 0.11 | 2.62 | 8.85×10^-4^ |
| chr7 | 150709571 | rs7830 | ATG9B | UTR3 | G/T | 0.05 | 2.05 | 2.47×10^-3^ |
| chr9 | 21166661 | rs8181184 | IFNA21 | upstream | C/T | 0.01 | 4.44 | 2.85×10^-3^ |
| chr15 | 66727597 | rs16949924 | MAP2K1 | intronic | G/C | 0.05 | 2.97 | 8.84×10^-4^ |
| chr17 | 34431403 | rs1719144 | CCL4 | intronic | G/A | 0.06 | 2.23 | 2.6×10^-3^ |
| chr19 | 2251512 | rs7252789 | AMH | exonic | T/A | 0.83 | 0.46 | 2.53×10^-3^ |
| chrY | 21153275 | rs6530599 | TTTY14 | ncRNA_intronic | A/G | 0.57 | 0.55 | 6.97×10^-4^ |

Ref/Alt, reference allele and alternative allele; OR, indicates odds ratio; and UTR, untranslated region
